# Cancer mortality in the United Kingdom: projections to the year 2025

**DOI:** 10.1038/sj.bjc.6604710

**Published:** 2008-10-14

**Authors:** A H Olsen, D M Parkin, P Sasieni

**Affiliations:** 1 Department of Mathematics and Statistics, Cancer Research UK Centre for Epidemiology, Mathematics and Statistics, Wolfson Institute of Preventive Medicine, Charterhouse Square, London EC1M 6BQ, UK

**Keywords:** malignant neoplasms, projections, predictions, trends, mortality

## Abstract

The purpose of this study was to project mortality rates in the United Kingdom for the period 2006–2025 for 21 major cancers on the basis of the observed trends in mortality rates during 1971–2005, and to estimate the implication in terms of expected deaths. Age-period-cohort models were applied to official statistics. The projected decrease in age-standardised mortality rates for all cancers from 2003 to 2023 was 17% in men and 16% in women. Future mortality rates were projected to decline for most cancer sites. In men, there were small projected increases in mortality rates from cancers of the oral cavity, oesophagus and melanoma, with a larger projected increase (14% over 20 years) in mortality of liver cancer. In women, the only projected increase (18%) was for corpus uteri. The numbers of deaths will increase for most cancers, with a 30% increase in all cancers projected for men and a 12% increase projected for women. Mortality rates from cancer as a whole have been falling in the United Kingdom since 1990, and this decline was projected to continue into the future as well as the declining rates in both sexes for most cancers. Actual numbers of deaths will increase for most cancers.

Projections of cancer incidence and mortality provide an estimate of the future burden of cancer and are fundamental to the process of planning for programmes of cancer control as well as for setting priorities for future research. In addition, by providing a benchmark of the expected rates of disease given current exposure to aetiological factors and healthcare interventions, projections become a valuable tool in evaluating the effectiveness of new programmes that aim to reduce incidence, mortality or both (Bray and Møller, 2006).

When predicting future incidence and mortality rates, it is necessary to be aware of the underlying assumptions and limitations. A distinction is sometimes made between projections and forecasts (Bashir and Estève, 2001). Projections derive from an extension into the future of past trends in rates of disease. By contrast, forecasts represent estimation of future rates taking into account expected changes in the factors determining the risk, or outcome, of the disease concerned.

In this report, we present projections of mortality rates of cancer in the United Kingdom to the period 2006–2025 for 21 major cancers on the basis of the observed trends in mortality rates during 1971–2005, and estimate the implication in terms of expected deaths, using government population projections.

## Materials and methods

The number of cancer deaths in the United Kingdom by cancer site, sex, 5-year age group and year for the 35-year period from 1971 to 2005, and population numbers for the same years and age–sex groups were provided by Cancer Research UK on the basis of separate national data from England, Scotland, Wales and Northern Ireland.

Population projections (2006 based) for the United Kingdom by sex, 5-year age group and year from 2006 to 2025 were downloaded from the Government Actuary's Department (2007).

The R-based software, ‘Nordpred’, developed at the Norwegian Cancer Registry for projections of nordic incidence and mortality rates (Møller *et al*, 2002), was used to project mortality rates in 2006–2025. The calculations are on the basis of the mortality rates from 1971 to 2005, aggregated into 5-year time periods from 1971–1975 to 2001–2005.

Future numbers of deaths were projected by multiplying the projected age-specific rates by the population projections for 2006–2025.

For projecting future rates the Nordpred software uses the age-period-cohort (APC) models, deriving the relevant parameters from the past observations and using them to estimate future rates. Here, the age, period and cohort function as pseudo indicators for factors that have influenced past trends, such as exposure to risk factors, treatment or screening affecting certain age groups, periods or cohorts. Within the basic APC model, the Nordpred software allows the choice of several options for modelling and projecting incidence and mortality rates. For this study, we used the model found by the Nordic research group to give the most accurate predictions of future numbers of 20 different cancer types (Møller *et al*, 2003). Thus, instead of using the standard exponential link function in poisson regression, the power of 5 was used. The reasoning behind this is that the power function levels of a potential exponential increase (so as to keep projections closer to the observed rates). The model can be written as 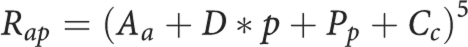 where *R*_*ap*_ is the mortality rate for age group *a* and period *p*, *A*_*a*_ is the age effect of age group *a*, *D* is the common linear drift of period and cohort, *P*_*p*_ is the non-linear period effect and *C*_*c*_ is the non-linear cohort effect, where *c*=*p*−*a*. Note that the 5-year periods are numbered consecutively so that *p* increases by 1 every 5 years; similarly, *a* counts 5-year age groups.

Those age groups for which less than 50 deaths were observed during any 5-year period were not included in any period in the modelling.

The age component was projected directly when estimated. For age groups not included in the model, the mean observed mortality for 1996–2005 was used for projections.

The linear drift *D* was projected into the future, but with two modifications. The so-called cut trend system was used, in the sense that instead of adding *D* for each future period, we added *D*, 3/4 *D*, 1/2 *D* and 1/4 *D* in the four future 5-year periods. The reasoning behind this was that whatever might have caused the increase or decrease in the past is not likely to continue unchanged indefinitely; rather some attenuation of such influences seems more plausible. In addition, Nordpred checks for significant curvature in the trends of the observed rates, and if this was present, the change in the last 10 years (recent slope), instead of the average change in the whole period (average slope), was used as the drift component in the future projections. The rationale is that the factors responsible for the most recent trends (when there has been a change) are more likely to be an influence on future rates, rather than those operative in the more distant past.

The non-linear cohort component was projected for estimated cohorts. For young cohorts not estimated, the last estimated cohort component was used.

The projections assume that the last non-linear period component applies to all future periods.

To estimate future rates for all cancers combined, the ‘other cancers’ category (i.e., other than the 21 sites studied) was created, and projected rates were calculated, as for the other sites. The total (‘all cancers’) was estimated as the sum of the numbers of deaths at the individual sites (including ‘other cancers’) divided by the population numbers.

The projected numbers of deaths were calculated using the projected mortality rates and the population projections from the Government Actuary's department (2007). In the tables, the numbers for 2023 are presented using the average population projections and the mortality rates for 2021–2025. The numbers for 2003 are presented using the average observed numbers for the corresponding 5-year period, 2001–2005.

Mortality rates are presented as age-standardised rates, according to the European standard population (Doll and Smith, 1982).

In 1984, the rules used to interpret secondary causes of death changed. In 1993, the rules from before 1984 were used again, and, in addition to this, the extent to which non-specific causes of death automatically generated medical enquiries changed. Estimates of the effects of these changes on disease-specific mortality rates have been published (Office of Population Censuses and Surveys, 1996). Age-specific mortality rates for the period 1971–1992 were adjusted to the coding practices from 1993 onwards using the factors in Table A.3 and B in the publication mentioned above (Office of Population Censuses and Surveys, 1996).

## Results

**Figure 1:**
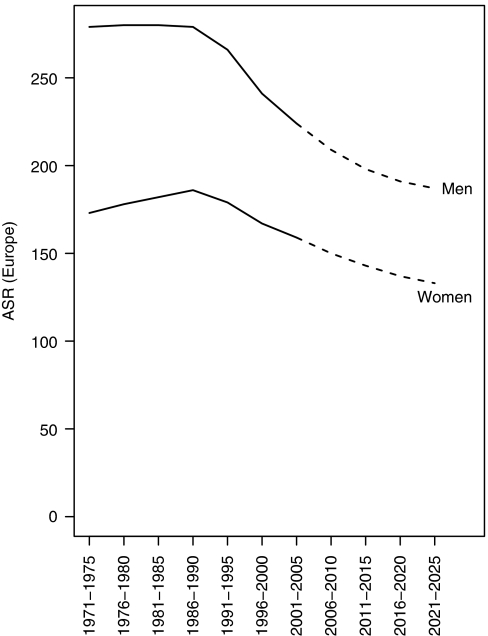
Trends and projections of the age-standardised (Europe) mortality rates for cancer (all sites except non-melanoma skin cancer) for men and women in the United Kingdom.

**Figure 2:**
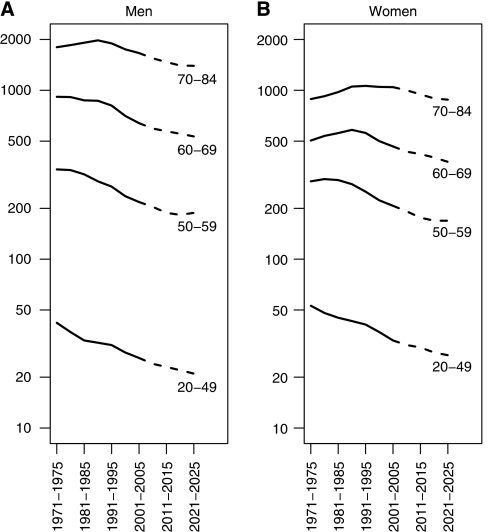
Age-specific trends and projections for cancer mortality rates (all sites except non-melanoma skin cancer) for men (**A**) and women (**B**) in the United Kingdom.

**Figure 3:**
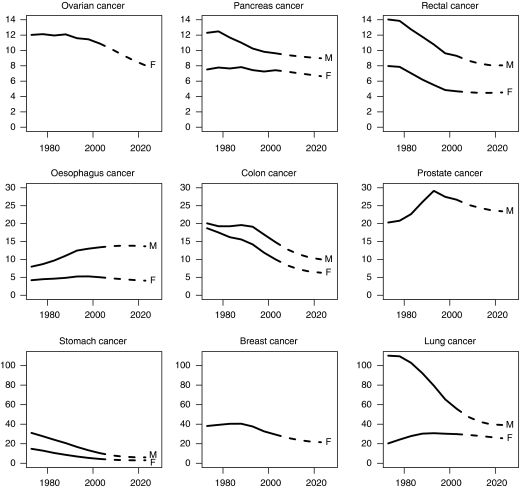
Trends and projections of the age-standardised (Europe) mortality rates for 21 different cancers for men (M) and women (F) in the United Kingdom.

Figure 1 shows the trends and projections of the age-standardised mortality rates for cancer (all sites) for men and women. Figure 2 shows the age-specific trends and projections for cancer mortality rates (all sites) for men (2A) and women (2B). Figure 3 shows trends and projections for 21 different cancers, by sex.

Table 1 shows, for men and women, the observed numbers of deaths by cancer site in 2003, and the numbers projected in 2023. The percentage change in the numbers of deaths is expressed as a percentage of the 2003 figures (% change overall), and this is divided into the component that results from the changes in mortality rates (risk) and the remainder, which is a consequence of demographic trends in the size and age composition of the population.

Mortality rates from cancer as a whole have been falling in the United Kingdom since 1990, and this decline is projected to continue into the future (Figure 1). For men as well as women, the decrease started earlier in the younger age groups indicating a cohort effect (Figure 2). The actual projected decrease in the age-standardised mortality rates between 2003 and 2023 is 17% in men and 16% in women. The projected increase in population size (59.6 million in 2003 and 68.0 million in 2023) and population ageing (11.5% aged 70 years or above in 2003, 14.7% in 2023) would result in an increase in the number of cancer deaths (by 57% in men and 31% in women), even if mortality rates remain unchanged. The combined effect of the increase because of demography and decrease because of declining rates is projected 30% increase in cancer deaths in men in the 20-year period and a 12% increase for women (Table 1).

Inspection of the trends and projections of mortality for the different cancers suggests declining future rates in both sexes for most cancers. In men, there are small projected increases in mortality rates from cancers of the oral cavity, oesophagus and melanoma, with a larger projected increase (14% over 20 years) in mortality from cancer of the liver. In women, only the age-adjusted mortality rate from cancer of the corpus uteri is projected to increase (by 18% over 20 years).

Despite these declines in mortality rates, the actual numbers of deaths will increase for most cancers, the exceptions being cancer of the stomach in men, and cancers of stomach, colon, larynx, cervix and breast in women.

## Discussion

Our projections of future mortality in the United Kingdom envisage declining rates of mortality for most cancer sites (and overall).

Future projections depend on multiple assumptions, but the basic premise is that past trends, affecting as they do the risk of death from cancer in specific generations and/or time periods, will be carried forward into the future. The Nordpred package allows for two additional assumptions — that recent trends are more relevant to the future evolution of risk than those in the distant past and that the impact of the factors underlying them (whatever they may be) will progressively diminish (the basis of the arbitrary reduction in the ‘drift’ component of the model in each 5-year period).

Projections do not take into account the impact of possible future changes in the incidence of cancer, or of dying from cancer, other than in a general way. They are, therefore, a useful basis for examining the possible scenarios resulting from the changes in policy with respect to prevention, screening or treatment (Black and Stockton, 2001; Parkin *et al*, 2008).

The projected numbers of deaths are dependant on reasonable population projections. In this study, we used the 2006-based population projections prepared by the Government Actuary's Department.

Møller *et al* (2007) carried out a similar analysis, using the same methodology, with respect to cancer incidence in England. In that study, 2004-based population projections were used. The projected population size in 2023 is 4% larger using the 2006-based compared with the 2004-based population estimates, although most of the differences concern the youngest and very oldest age groups. The variation at ages 50–79 (where 60% of cancer deaths occur) is small (<1%). Although incidence rates in men were projected to decline by 7% between 2001 and 2020 (driven largely by the declining rates of lung cancer), female rates were projected to increase by the same amount, with significant increases in the risk of breast cancer in particular. The differences between the projections of mortality and those of incidence (other than the somewhat different populations studied – the United Kingdom and England, respectively) are in part explained by the improvement in prognosis for some cancers in recent years, partly as a result of screening and partly because of better treatment. Thus, in England, survival rates have increased between 1986 and 1999 for several major cancers – at 1% annually for cancers of the breast and colon-rectum, around 0.6–0.8% annually for cancers of the ovary, kidney, melanoma, leukaemia and lymphoma and by 2.3% for cancer of the prostate (Cancer Research UK, 2007). This is particularly obvious for cancer of the breast – the second most common cancer in United Kingdom – for which incidence rates have, until very recently, been increasing, whereas mortality has declined since 1988 (Cancer Research UK, 2008). Similarly, the incidence of prostate cancer in men in the United Kingdom increased by 4.1% annually since 1994, whereas mortality has declined by 1.1% a year (Cancer Research UK, 2008).

In this study, we have projected cancer mortality rates 20 years into the future. Projections are more likely to be close to reality in the near future. However, programmes of early detection and, even more so, prevention will not influence mortality rates for many years after their introduction. The projections in this paper form the basis for research into the effect of various scenarios for interventions such as these, as well as improvements in treatment and the delivery of cancer services, so that a period of at least 20 years is necessary to fully evaluate their potential effects.

## Figures and Tables

**Table 1:** Observed and projected numbers of deaths by cancer site and projected change in mortality for (a) men and (b) women in the United Kingdom

**ICD10**	**Cancer type**	**Deaths 2003**	**Deaths 2023**	**Overall change (%)**	**Change in risk (%)**	**Additional change**
(a)						
C00-06, C09-10, C12-14	Oral	1100	1594	45	3	42
C15	Oesophagus	4657	7114	53	2	51
C16	Stomach	3748	3275	−13	−43	31
C18	Colon	5317	5838	10	−32	42
C19-21	Rectum	3276	4387	34	−13	47
C22	Liver	1520	2537	67	14	53
C25	Pancreas	3367	4763	41	−6	48
C32	Larynx	667	743	11	−25	37
C33-34	Lung	19 866	20 542	3	−31	34
C43	Malignant melanoma	927	1449	56	8	48
C64-66	Kidney	2114	2864	35	−11	46
C61	Prostate	10 039	15 469	54	−12	67
C67	Bladder	3203	3776	18	−32	49
C71	Brain and other CNS	1984	2327	17	−11	29
C82-85	Non-Hodgkin's lymphoma	2410	2886	20	−23	42
C88, C90	Multiple myeloma	1314	1982	51	−4	55
C91-95	Leukaemia	2375	3674	55	−3	58
C00-97 (excluding C44)	All cancer except non-melanoma skin cancer	79 801	103 626	30	−17	47
						
(b)						
C00-06, C09-10, C12-14	Oral	571	746	31	0	31
C15	Oesophagus	2593	2875	11	−17	28
C16	Stomach	2328	2008	−14	−36	22
C18	Colon	5190	4470	−14	−36	22
C19-21	Rectum	2363	2895	22	−8	31
C22	Liver	1076	1426	33	0	32
C25	Pancreas	3626	4378	21	−9	30
C32	Larynx	171	151	−12	−32	20
C33-34	Lung	13 521	15 211	12	−14	26
C43	Malignant melanoma	803	829	3	−22	25
C50	Breast	12 642	11 834	−6	−28	21
C53	Cervix	1110	609	−45	−57	11
C54	Corpus uteri	1093	1716	57	18	39
C56-57	Ovary	4563	4694	3	−21	24
C64-66	Kidney	1325	1587	20	−9	29
C67	Bladder	1669	1752	5	−23	28
C71	Brain and other CNS	1425	1439	1	−21	22
C82-85	Non-Hodgkin's lymphoma	2176	2205	1	−24	25
C88,C90	Multiple myeloma	1231	1460	19	−11	30
C91-95	Leukaemia	1905	2277	20	−11	30
C00-97 (excluding C44)	All cancer except non-melanoma skin cancer	73 909	82 415	12	−16	27
